# Sequelae of Typho-Malarial Fever, Complicated with Early State of Opium Habit Established by Medical Attendants

**Published:** 1899-11

**Authors:** Chas. J. Vaughan

**Affiliations:** Assistant Professor to Chair of Obstetrics and Gynecology, Atlanta College of Physicians and Surgeons, Atlanta, Ga.


For Texas Medical Journal.

### Sequelae of Typho-Malarial Fever, Complicated with Early State of Opium Habit Established by Medical Attendants.

BY CHAS. J. VAUGHAN, M. D.,
Assistant Professor to Chair of Obstetrics and Gynecology, Atlanta College of Physicians and Surgeons, Atlanta, Ga.

Bearing in mind the influence of climate and soil upon pathological conditions, not forgetful of isothermal effects relative to geography and topography, and the modification of treatment imposed by environment, I am led to report some clinical notes I have recently made of a case which may interest your readers.

When I was first called to see Mrs. L., her husband gave me the following history: Age 32, native of southeast Georgia; she had lived in Florida twelve months previous to a six months residence in this city, to which place her husband brought her for change of climate.

Nine months before I was consulted patient had severe attack of typho-malarial fever attended with hemorrhages of the bowels; was a great sufferer from malaria for several years. Had one miscarriage, which caused laceration of the os and perineum.

During six months residence in this city had consulted and received "treatment" from several quacks of itinerant persuasion who "guaranteed a cure at thirty dollars a month."

I found the patient in bed, dorsal position, greatly emaciated, unable to speak above a hoarse whisper, extremities cold and benumbed, sallowness of complexion unusually marked, facial expression dull and listless, eyes leaden, pupils contracted, lips livid and relaxed. Complained of chronic constipation and frequent attacks of colic. Menstrual functions irregular, painful and profuse. Unable to sleep without recourse to "medicine left by the doctor," which medicine had been used up the night before, and the "doctor" had "done gone and left the city." Heart weak and irregular, temperature elevated three and two-fifths degrees; tongue thickly coated dark brown. General appearance of affairs grave.

Naturally, my efforts were directed toward gentle stimulation of heart and emunctories, reduction of temperature, use of mild hypnotic, alimentary and systemic tonics, antiperiodics and reconstituants.

The husband warned me against using purgatives "such as calomel and pills," which, he assured me, had no effect on his wife except to make "her mouth sore and teeth loose." Castor oil he said "worked sometimes and then again it didn't."

I prescribed very moderate doses of digitalis, nux vomica, and liquor pot. ars. (Fowler's sol.), but was not a little perplexed to meet the other requirements satisfactorily. However, they were carefully considered, and as subsequent developments attest, ideally met in "antikamnia and quinine laxative tablets." Each tablet of this new compound consisting of Antikamnia, gr. 3; quin. bi-sulph., gr. $1\frac{3}{4}$; Cascarin, gr. $\frac{1}{8}$; Aloin, gr. 1-32; extr. Belladon., gr. 1-32; Podophyllin, gr. 1-32. I prescribed one tablet every hour. This I ordered continued until the patient should fall asleep, which she did after six tablets had been thus administered. Her rest was much broken, however.

I called the following morning, and though the temperature had fallen 1⅛ degrees, there were few signs of improvement—a condition not unexpected. I ordered the tablets continued every hour, as before, until my next visit, which was six hours later. The temperature by this time had fallen another degree, and the patient had had a free evacuation of the bowels, was resting comfortably, and appeared decidedly improved. After this the tablets were continued, one every three hours, while the patient was awake. On the third day I discontinued the arsenic and nux vom., as the former was evidently causing a slight derangement of the stomach; which was weak, empty and irritable; and the latter seemed disturbing to the patient's nerves. The pyrexia was yielding beautifully to the treatment, peristalsis gradually reëstablished, the emunctories returning to approximate functional activity, and on the fifth day I found no further occasion to use the digitalis. I continued the Antikamnia and Quinine Laxative Tablets, however, and on the sixth day lengthened the interval between doses one hour. This was continued for five days longer, during which time special attention was paid to dietary regime. On the eleventh day patient was sitting up. Thereafter I ordered one tablet four times a day; *i. e.*, one hour before each meal and one at bed time.

On the fifteenth day I discharged patient, who said she was feeling better than she had felt in two years. She had rapidly put on flesh—complexion very noticeably clearer—mucous surfaces inclined to red—appetite fairly good.

